# Density of human bone marrow stromal cells regulates commitment to vascular lineages

**DOI:** 10.1016/j.scr.2011.02.001

**Published:** 2011-05

**Authors:** Jemima L. Whyte, Stephen G. Ball, C. Adrian Shuttleworth, Keith Brennan, Cay M. Kielty

**Affiliations:** Wellcome Trust Centre for Cell-Matrix Research, Faculty of Life Sciences, University of Manchester, UK

**Keywords:** αSMA, smooth muscle alpha actin, DAPT, *N*-[*N*-(3,5-difluorophenacetyl-L-alanyl)]-*S*-phenylglycine *t*-butyl ester, DMSO, dimethyl sulfoxide, ECs, endothelial cells, EDTA, ethylenediaminetetraacetic acid, HES-1, hairy enhancer of split-1, HUVECs, human umbilical vein endothelial cells, LDL, low density lipoprotein, HBMSCs, human bone marrow stromal cells, PECAM-1, platelet endothelial cell adhesion molecule-1 (CD31), SM-MHC-1, vascular smooth muscle myosin heavy chain 1, VCAM-1, vascular cell adhesion molecule, VE-cadherin, vascular endothelial cadherin, VEGF, vascular endothelial growth factor, VEGFR1, vascular endothelial growth factor receptor 1, VEGF-I, VEGF neutralization antibody, VEGFR-I, VEGFR signaling inhibitor, vSMC, vascular smooth muscle cell, vWF, Von Willebrand factor.

## Abstract

Mechanisms underlying the vascular differentiation of human bone marrow stromal cells (HBMSCs) and their contribution to neovascularisation are poorly understood. We report the essential role of cell density-induced signals in directing HBMSCs along endothelial or smooth muscle lineages. Plating HBMSCs at high density rapidly induced Notch signaling, which initiated HBMSC commitment to a vascular progenitor cell population expressing markers for both vascular lineages. Notch also induced VEGF-A, which inhibited vascular smooth muscle commitment while consolidating differentiation to endothelial cells with cobblestone morphology and characteristic endothelial markers and functions. These mechanisms can be exploited therapeutically to regulate HBMSCs during neovascularisation.

## Introduction

Human bone marrow stromal cells (HBMSCs) have therapeutic potential in cell transplantation and tissue engineering. In addition to their anti-inflammatory and immunomodulatory characteristics (Lee et al., 2009a, Casiraghi et al., 2010), they have the capacity to differentiate directly along skeletal and other mesenchymal lineages (Pittenger et al., 1999, Augello and De Bari, 2010). A major challenge now is to define the mechanisms that regulate their differentiation along vascular endothelial cell (EC) and smooth muscle cell (vSMC) lineages.

Cultured HBMSCs typically possess some characteristics of synthetic proliferative vSMCs. In particular, they express the early contractile marker smooth muscle alpha actin (αSMA), as well as calponin and smoothelin-B (Ball et al., 2004). Stimuli shown to induce the differentiation of HBMSCs towards vSMCs include mechanical strain (Kurpinski et al., 2006), inhibition of DNA methylation (Wakitani et al., 1995), and transforming growth factor (TGF)β and Notch signaling (Kurpinski et al., 2010). Other studies have indicated that HBMSCs have the potential to differentiate towards EC lineages (Al-Khaldi et al., 2003, Bai et al., 2009, Chen et al., 2009, Chung et al., 2009, Lozito et al., 2009, Ohata et al., 2009, Oswald et al., 2004, Siekmann et al., 2008, Wu et al., 2005a, Xu et al., 2009, Yue et al., 2008, Zhang et al., 2008). In many of these studies, HBMSCs exposed over days to exogenous vascular endothelial growth factor (VEGF-A) showed evidence of differentiation towards ECs. However, HBMSCs do not always express vascular endothelial growth factor receptors (VEGFRs) (Chen et al., 2009, Oswald et al., 2004, Ball et al., 2007). Notch signaling has also been shown to direct HBMSCs towards an EC fate when activated experimentally using recombinant ligands, chemical exposure, or expression constructs (Ohata et al., 2009, Siekmann et al., 2008, Xu et al., 2009, Zhang et al., 2008).

Notch and VEGF signaling coordinate endothelial cell activity during vascular development, arterio-venous specification, vessel branching and endothelial tip cell formation (Gridley, 2007, Lawson et al., 2001, Boulton et al., 2008, Roukens et al., 2010, Jakobsson et al., 2010). We have investigated how these signaling mechanisms also direct the commitment of HBMSCs toward vascular ECs or vSMCs. We report that plating HBMSCs at high density rapidly stimulates Notch signaling, which induces vascular progenitor cells with the capacity to give rise to either EC or vSMC lineages. VEGF-A is not essential for the initiation of HBMSC differentiation to ECs but regulates the fate decision between EC and vSMC lineages. These mechanistic insights may be exploited in therapeutic applications of HBMSCs.

## Results

### HBMSCs plated and cultured at high density differentiated toward endothelial cells

**Figure 1:**
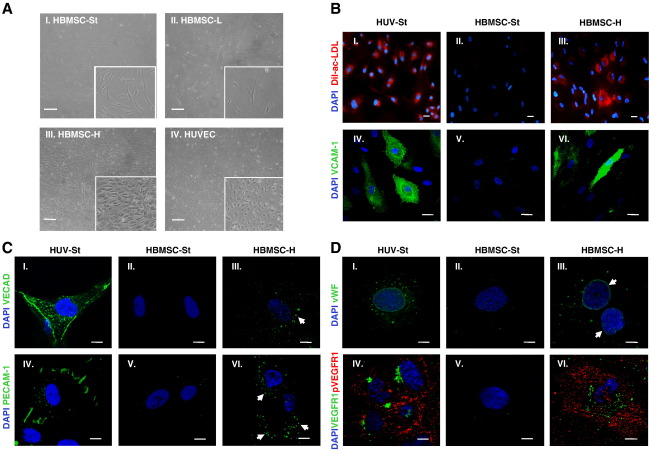
HBMSCs plated and cultured at high density differentiated toward functional ECs. To determine whether HBMSCs plated and cultured at high density differentiated toward EC over time, their morphology, EC functional properties, and expression of EC markers were determined. (A) Representative phase contrast images of (I) HBMSCs under standard culture conditions (MSC-St) for 48 h, (II) HBMSCs cultured at low density (HBMSC-L) for 14 days, or (III) HBMSCs cultured at high density (HBMSC-H) for 14 days. (IV) HUVECs under standard culture conditions were used as a positive cellular control. For culture conditions, see Materials and methods. Images were obtained using an Olympus (CK X41) microscope (4x objective). Inserts represent an enlarged region of each image. Scale bars = 200 μm. (B) (I–III) Immunofluorescence analysis showing DiI-Ac-LDL uptake in (I) HUVECs, (II) HBMSCs under standard culture conditions (HBMSC-St) for 48 h, and (III) HBMSCs cultured at high density (HBMSC-H) for 28 days. DiI-Ac-LDL uptake (red), DAPI (blue). Images obtained using an Olympus upright widefield fluorescence (BX51) microscope (20x objective). Scale bars = 20 μm. (IV–VI) Immunofluorescence analysis of VCAM-1 in cells stimulated with 10 μg/ml TNFα for 24 h. (IV) HUVECs under standard culture conditions for 48 h, (V) HBMSCs under standard culture conditions (HBMSC-St) for 48 h, and (VI) HBMSCs at high density (HBMSC-H) for 28 days. VCAM-1 (green), DAPI (blue). Images were obtained using a Nikon C1 upright confocal microscope (60x objective). Scale bars = 20 μm. (C,D) Immunofluorescence analysis of VE-cadherin, PECAM-1, vWF, and VEGFR1 expression in (I, IV) HUVECs under standard culture conditions for 48 h, (II, V) HBMSCs under standard culture conditions for 48 h, or (III, VI) HBMSCs cultured at high density (HBMSC-H) for (C) 28 days or (D) 14 days. VE-cadherin, PECAM-1, vWF, and VEGFR1 (green), phosphorylated VEGFR1 (red), DAPI (blue). Images were obtained using a Nikon C1 upright confocal microscope (60x objective) (C, I–VI; and D, IV–VI) or using an Olympus IX71 Deltavision microscope (40x objective) (D, I–III). Scale bars = 10 μm. White arrows highlight specific regions of localised immunoreactivity. Representative images from two independent experiments.

Cell density can regulate the morphology and differentiation of HBMSCs (Ball et al., 2004, McBeath et al., 2004). Using HBMSCs which could be induced to differentiate toward adipocytes, osteocytes, or chondrocytes (Supplementary Fig. S1), we studied the impact of plating HBMSCs at high cell density on their differentiation toward ECs, monitoring morphology (Fig. 1A), specific functional properties (Fig. 1B), and expression of a panel of characteristic endothelial markers (Figs. 1C and D).

HBMSCs plated at high density and cultured for 14 days (Fig. 1AIII) adopted a cobblestone-like morphology comparable to that of human umbilical vein endothelial cells (HUVECs) (Fig. 1AIV), whereas HBMSCs cultured under standard conditions for 48 h (Fig. 1AI), or at low cell density for 14 days (Fig. 1AII), maintained a spindle-shaped morphology.

HBMSCs plated at high density and cultured for 28 days displayed low density lipoprotein (LDL) uptake in approximately 53% of cells analysed (Fig. 1BIII), and VCAM-1 was also induced in response to TNFα (Fig. 1BVI). In contrast, HBMSCs cultured under standard conditions for 48 h did not display LDL uptake (Fig. 1BII) or VCAM-1 induction in response to TNFα (Fig. 1BV). As expected, HUVECs as positive control cells exhibited EC functional characteristics of LDL uptake (Fig. 1BI) and induction of VCAM-1 in response to TNFα (Fig. 1BIV).

The distribution of the EC markers VE-cadherin, PECAM-1, vWF, and VEGFR1 in HBMSCs was examined by immunofluorescence microscopy. HBMSCs plated at high density and cultured for 14 or 28 days displayed prominent EC marker immunostaining (Figs. 1CIII and VI, DIII and VI). In contrast, HBMSCs cultured under standard conditions for 48 h displayed little or no immunofluorescence for the EC markers examined (Figs. 1CII and V, DII and V). As expected, HUVECs as positive control cells expressed the characteristic markers VE-cadherin (Fig. 1CI), PECAM-1 (Fig. 1CIV), vWF (Fig. 1DI), and VEGFR1 (Fig. 1DIV).

### High density HBMSCs expressed endothelial and smooth muscle cell markers by 24 h

As plating at high cell density for up to 28 days induced HBMSCs to adopt an endothelial phenotype (Fig. 1), we further investigated the time scale of these cellular changes by examining the expression of vascular cell markers following HBMSC culture at high density for 24 h and 3 days. The expression of EC (vWF, VEGFR1 and PECAM-1) (Figs. 2A–C) and vSMC (αSMA, calponin, smoothelin-B, and SM-MHC-1) (Figs. 2D–G) markers were analysed by immunoblot analysis. Compared to HBMSCs cultured at low density for 24 h (lane 1), culturing HBMSCs at high density (lane 2) not only induced the expression of the EC markers vWF (Fig. 2A), VEGFR1 (Fig. 2B), and PECAM-1 (Fig. 2C), but markedly up-regulated the expression of vSMC markers α-SMA (Fig. 2D), calponin (Fig. 2E), smoothelin-B (Fig. 2F), and SM-MHC-1 (Fig. 2G). To determine whether individual HBMSCs were expressing both EC and vSMC markers, double-labeling immunofluorescence analysis was conducted (Fig. 2H). This analysis clearly demonstrated that individual HBMSCs were coexpressing EC and vSMC markers following culture at high cell density for 24 h. Thus, both EC and vSMC markers were enhanced within 24 h of plating HBMSCs at high density, indicating a vascular progenitor phenotype with potential to form ECs or vSMCs.

**Figue 2:**
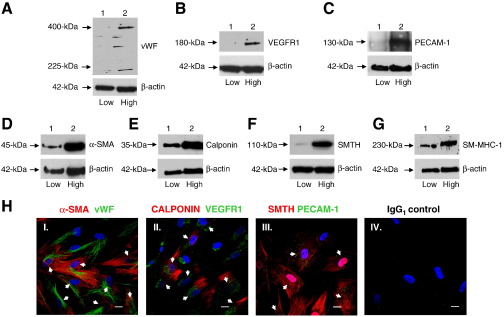
HBMSCs plated at high density and cultured for 24 h expressed EC and vSMC markers. HBMSCs plated and cultured at high density for 24 h were examined for the expression of vascular cell markers. (A–G) Immunoblot analysis of HBMSCs cultured at high density for 24 h to determine the expression of markers for EC and vSMC. (A) vWF, (B) VEGFR1, (C) PECAM-1, (D) αSMA, (E) calponin, (F) smoothelin-B (SMTH), and (G) SM-MHC-1 in HBMSCs cultured at low density for 24 h (lane 1) or at high density for 24 h (lane 2). Membranes were reprobed with β-actin as loading controls. PECAM-1 was immunoprecipitated prior to immunoblotting and equal volumes of protein lysate were immunoblotted for β-actin as loading controls. Representatives of two independent experiments are shown for each analysis. (H) Double-labeling immunofluorescence analysis to detect individual HBMSCs coexpressing EC and vSMC markers following culture at high cell density for 24 h. vSMC markers (I) αSMA, (II) calponin, and (III) smoothelin-B (red); EC markers (I) vWF, (II) VEGFR1, and (III) PECAM-1 (green); DAPI (blue). The isotype control IgG_1_ is also depicted. Images were obtained using a Nikon C1 upright confocal microscope (60x objective). Scale bars represent 20 μm. White arrows highlight individual HBMSCs coexpressing EC and vSMC markers. Representative images obtained from two independent experiments are shown.

### High density HBMSCs enhanced endothelial markers but down-regulated smooth muscle cell markers by 3 days

**Figure 3:**
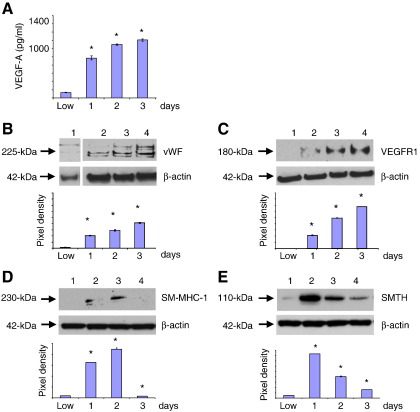
High density HBMSCs expressed increased endothelial cell markers but reduced smooth muscle cell markers, by 3 days. The levels of vascular cell markers were determined in HBMSCs plated and cultured at high density for 3 days. (A) ELISA assay of VEGF-A derived from medium taken from HBMSCs cultured at low density for 24 h, or at high cell density up to 3 days, normalised against corresponding β-actin levels. (B–E) Immunoblot analysis of (B) vWF, (C) VEGFR1, (D) SM-MHC-1, and (E) smoothelin-B (SMTH) in HBMSCs cultured at low density (HBMSC-L) for 24 h or at high cell density up to 3 days. Pixel density was normalised to β-actin and plotted as bar graphs. * *P* < 0.05 compared to HBMSCs cultured after plating at low cell density.

HBMSC expression of vascular markers was further examined after 3 days. Compared to HBMSCs cultured at low density, the level of VEGF-A secreted by HBMSCs at high cell density, normalised against corresponding β-actin levels, was significantly increased over a 3-day period (Fig. 3A). This enhanced VEGF-A secretion also coincided with a significant increase in the expression of EC markers (Figs. 3B and C); however, in comparison, expression of vSMC markers SM-MHC-1 (Fig. 3D) and smoothelin-B (Fig. 3E) dramatically decreased by 3 days. Thus, sustained culture of HBMSCs plated at high density promoted EC differentiation but inhibited vSMC differentiation.

### Notch signaling induced vascular progenitor cells and VEGF-A secretion

**Figure 4:**
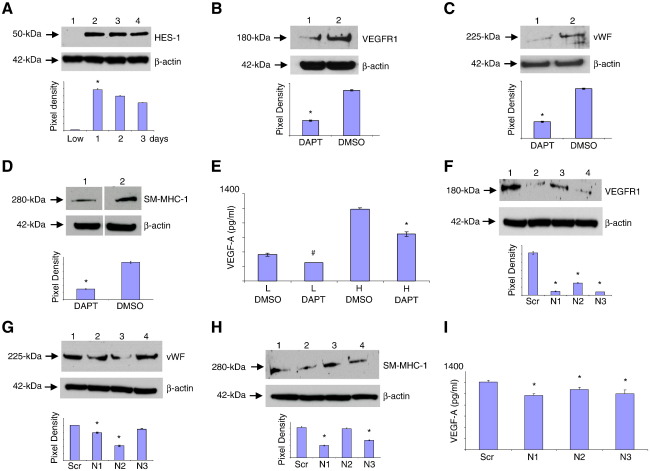
Notch signaling stimulated the formation of the vascular progenitor cell and VEGF-A secretion. The role of Notch signaling in regulating the expression of vascular cell markers in HBMSCs at high cell density was determined. (A) Immunoblot analysis of HES-1 in HBMSCs cultured at low density (lane 1) for 24 h or at high density up to 3 days (lanes 2–4). (B–D) To investigate whether Notch signaling regulated the expression of vascular cell markers and VEGF-A, their protein levels were determined following inhibition of Notch signaling by DAPT treatment (50 μM), with DMSO used as a diluent control. Immunoblot analysis of (B) VEGFR1, (C) vWF, or (D) SM-MHC-1 from high density HBMSCs cultured in the presence of DAPT (lanes 1) or DMSO (lanes 2). Pixel density was normalised to β-actin and plotted as bar graphs. * *P* < 0.05 compared to HBMSCs plated at high density in the presence of DMSO only. (E) ELISA assay of VEGF-A derived from the medium taken from HBMSCs cultured at low cell density (L) or high cell density (H) in the presence of DMSO as a diluent control, or DAPT, normalised against corresponding β-actin levels. * *P* < 0.05 compared to HBMSCs cultured after plating at high cell density in the presence of DMSO only. (F–I) To confirm that Notch signaling induced vascular marker expression and VEGF-A secretion, siRNA knockdown of Notch receptors 1, 2, or 3 (N1–N3) was performed. Immunoblot analysis of (F) VEGFR1, (G) vWF, and (H) SM-MHC-1 from HBMSCs cultured for 24 h after plating at high cell density, following transfection with scrambled (Scr) controls (lane 1) or siRNAs for Notch receptors 1, 2, and 3 (lanes 2–4). Membranes were reprobed with β-actin as loading controls. Pixel density was normalised to β-actin and plotted as a bar graph. * *P* < 0.05 compared to scrambled control-transfected HBMSCs. Data are representative of two independent experiments. (I) ELISA assay of VEGF-A derived from medium taken from HBMSCs cultured at high cell density following Notch receptor siRNA knockdown, normalised against corresponding β-actin levels. * *P* < 0.05 and # *P* < 0.1 compared to scrambled control HBMSCs.

Notch signaling is known to regulate vascular remodeling (Gridley, 2007). To investigate whether signaling through Notch receptors controlled the density-dependent differentiation of HBMSCs toward vascular phenotypes, Notch signaling molecules were examined up to 3 days after plating HBMSCs at high density (Fig. 4). The Notch transcription factor HES-1 was not detected in HBMSCs cultured at low cell density, but was rapidly induced within 24 h of plating HBMSCs at high density, and levels remained high during 3 days culture (Fig. 4A). To investigate whether Notch signaling may contribute to inducing vascular progenitor cells within 24 h, HBMSCs plated at low density or high density were immunostained for Notch receptors 1–4. Only the high density HBMSCs strongly expressed Notch receptors 1, 2, and 3, (Supplementary Fig. S2), while Notch receptor 4 was not detected (not shown). This result was confirmed by qPCR and immunoblot analysis (not shown).

DAPT is a chemical inhibitor of Notch signaling that inhibits the transcription of target genes, including HES-1 (Boulton et al., 2008) (Supplementary Fig. S3A). HBMSCs plated at high density were therefore treated with DAPT for 24 h, and then the expression of markers for EC and vSMC were determined by immunoblot analysis (Figs. 4B–D). HBMSCs plated at high density and cultured in the presence of DAPT showed a significant decrease in VEGFR1 and vWF (Figs. 4B and C) and SM-MHC-1 (Fig. 4D) compared to DMSO-treated control HBMSCs. In addition, the level of VEGF-A secreted by DAPT-treated HBMSCs at high density for 24 h, normalised against corresponding β-actin levels, was also significantly inhibited (Fig. 4E). Thus, Notch signaling enhanced VEGF-A secretion and induced a vascular progenitor cell state.

To examine whether Notch receptors 1–3 were involved in inducing vascular progenitor cells and stimulating VEGF-A secretion, siRNA knockdown was performed. The efficacy of each siRNA knockdown compared to scrambled siRNAs was confirmed by immunoblot analysis (Supplementary Fig. S3B). The specificity of each siRNA Notch receptor knockdown was confirmed by reprobing the blots for a nontarget Notch receptor, which revealed unchanged protein levels in each case (Supplementary Fig. S3B). Knockdown of Notch receptors was therefore used to investigate which Notch receptors were involved in regulating the expression of vascular markers in HBMSCs plated at high density for 24 h (Figs. 4F–I). Knockdown of Notch receptor 1, 2, or 3 demonstrated a significant decrease both in VEGFR1 expression (Fig. 4F) and in VEGF-A secretion (Fig. 4I). Knockdown of Notch receptor 1 or 2 significantly inhibited vWF expression (Fig. 4G, lanes 2 and 3), while knockdown of Notch receptors 1 or 3 significantly inhibited SM-MHC-1 expression (Fig. 4H, lanes 2 and 4).

Thus, Notch signaling activated by plating HBMSCs at high cell density is required for induction of vascular progenitor cells and commitment toward ECs.

### Notch activation stimulated HBMSCs at low density to express EC markers

**Figure 5:**
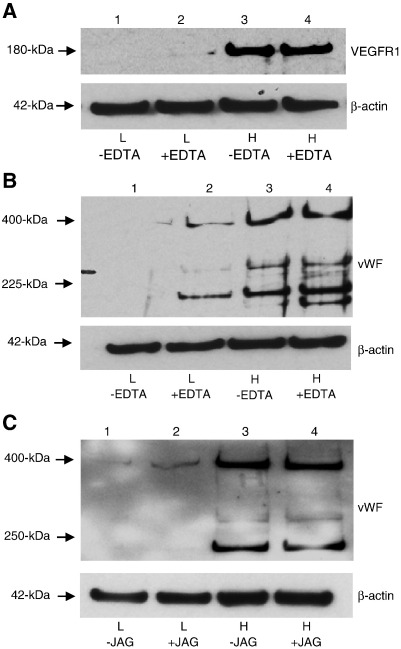
Notch activation stimulated HBMSCs at low density to express EC markers. To determine whether experimental activation of Notch could induce vWF and VEGFR1 protein expression in HBMSCs plated at low density and cultured for 24 h, HBMSCs were treated with Notch activators. (A,B) Immunoblot analysis of (A) VEGFR1 or (B) vWF protein expression from HBMSCs cultured after plating at low density (L) (lanes 1 and 2), or high cell density (H) (lanes 3 and 4), in the absence (−) (lanes 1 and 3) or presence (+) (lanes 2 and 4) of 5 mM EDTA. (C) To verify that HBMSCs cultured at low density could be induced to up-regulate vWF protein expression by activation of Notch signaling, Notch was activated using 5 μM Jagged-1. Immunoblot analysis of vWF protein expression in HBMSCs cultured after plating at low density (lanes 1 and 2) or high cell density (lanes 3 and 4) in the absence (−) (lanes 1 and 3) or presence (+) (lanes 2 and 4) of 5 μM Jagged-1. Membranes were reprobed with β-actin as loading controls. Data are representative of two independent experiments.

We examined whether activation of Notch signaling could induce the vascular potential of HBMSCs plated and cultured at low cell density. Notch signaling can be activated by treating cells with cation chelators, which cause the rapid shedding of the Notch extracellular domain, thereby increasing Notch receptor 1 intranuclear staining and transcription of a nuclear mediator of Notch signaling (Rand et al., 2000, Aster et al., 1999, Rand et al., 1997). HBMSCs plated at high density and cultured for 24 h in the presence of 5 mM EDTA displayed an increase in HES1, demonstrating the efficacy of EDTA treatment (Supplementary Fig. S3C). While neither VEGFR1 nor vWF were detected when HBMSCs were plated at low cell density and cultured for 24 h in the absence of EDTA (Figs. 5A and B, lanes 1), HBMSCs cultured at low cell density in the presence of EDTA expressed both VEGFR1 (Fig. 5A, lane 2) and vWF (Fig. 5B, lane 2). Thus, activation of Notch signaling by EDTA exposure induced the expression of EC markers that would otherwise only be expressed by HBMSCs plated at high density.

To confirm that HBMSCs plated at low density and cultured for 24 h could be induced to up-regulate expression of vWF by Notch activation, HBMSCs at low cell density were exposed to the Notch ligand Jagged-1 (Fig. 5C). Immunoblot analysis revealed that, compared to unstimulated HBMSCs (Fig. 5C, lane 1), exposure to Jagged-1 induced HBMSCs at low cell density to enhance vWF expression (Fig. 5C, lane 2).

Thus, Notch signaling was sufficient to stimulate EC marker expression in HBMSCs at low cell density.

### VEGF-A promoted EC differentiation but inhibited vSMC differentiation

**Figure 6:**
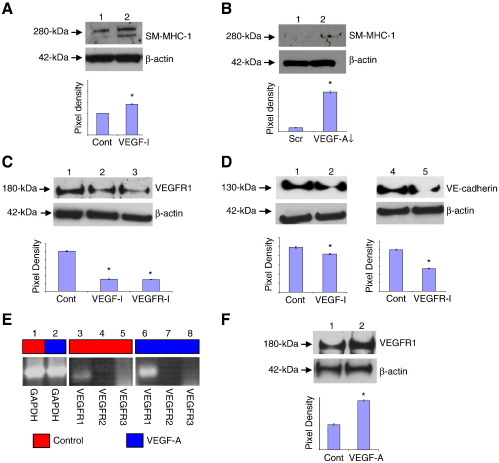
VEGF-A inhibited HBMSC differentiation to vSMCs but promoted EC differentiation. To determine whether secreted VEGF-A inhibited vSMC marker expression in HBMSCs cultured at high density, the effects of blocking VEGF-A signaling was determined. (A,B) HBMSCs cultured at high density for 24 h were (A) exposed to 1 μg/ml VEGF neutralisation antibody (VEGF-I) (lane 2) or (B) transfected with VEGF-A siRNA (lane 2), and then SM-MHC-1 expression was determined by immunoblot analysis. Untreated HBMSCs (Cont) or scrambled (Scr) siRNA-transfected HBMSCs (lane 1) were used as respective controls. Pixel density was normalised to β-actin and plotted as a bar graph. * *P* < 0.05 compared to controls. Data are representative of two independent experiments for each analysis. (C,D) HBMSCs cultured at high density for 14 days were exposed to 1 μg/ml VEGF-I (lane 2) or 0.5 μM VEGFR tyrosine kinase inhibitor (VEGFR-I) (C, lane 3, and D, lane 5), and then (C) VEGFR1 and (D) VE-cadherin expression was determined by immunoblot analysis. Untreated HBMSCs were used as controls (Cont) (C, lane 1, and D, lanes 1 and 4). Pixel density was normalised to β-actin and plotted as a bar graph. * *P* < 0.05 compared to untreated HBMSCs. A representative of two independent experiments is shown in each case. (E) HBMSCs cultured at high density for 14 days were exposed to 50 ng/ml recombinant VEGF-A, and then VEGFR transcript expression was determined by semiquantitative RT-PCR analysis. Lanes 1–2, GAPDH in untreated (control) HBMSCs. VEGFRs 1–3 in untreated (control) HBMSCs, lanes 3–5, or following exposure to recombinant VEGF-A, lanes 6–8, respectively. Two different primer pairs for VEGFRs 1–3 gave similar results. Red boxes denote unstimulated cells; blue boxes denote VEGF-A-stimulated cells. (F) Immunoblot analysis of VEGFR1 protein level in untreated HBMSCs (lane 1), or following exposure to recombinant VEGF-A for 14 days (lane 2). * *P* < 0.05 compared to untreated HBMSCs cultured after plating at high cell density (HBMSC-H). In each case, fresh medium supplemented with VEGF-A or inhibitors was added to cells every 2 days.

Since HBMSCs plated at high density for 24 h significantly increased their VEGF-A secretion, which was Notch dependent (Fig. 4E), experiments were conducted to determine if Notch-induced VEGF-A inhibited differentiation toward vSMCs. When HBMSCs that had been plated at high cell density and cultured for 24 h in the presence of a VEGF neutralisation antibody (designated VEGF-I) (Supplementary Fig. S3D), there was a significant up-regulation of SM-MHC-1 (Fig. 6A) and desmin expression (data not shown). To confirm that VEGF-A suppressed SM-MHC-1 expression, VEGF siRNA knockdown was performed (Supplementary Figs. S3E and F). VEGF-A knockdown was shown significantly to up-regulate SM-MHC-1 (Fig. 6B), indicating that VEGF-A was inhibiting expression of this definitive vSMC contractile marker.

VEGF-A has been implicated in directing HBMSC differentiation toward ECs (see Introduction). However, exposure to exogenous VEGF-A for 24 h did not initiate HBMSC commitment to ECs (Supplementary Figs. S4A–C). To investigate whether, over a longer time frame, VEGF-A influenced HBMSC commitment to ECs, HBMSCs were plated at high density and cultured for 14 days in the absence or presence of VEGF-I, or a chemical inhibitor of VEGF signaling (designated VEGFR-I) (Supplementary Fig. S3G). In the presence of VEGF-I or VEGFR-I, HBMSCs showed a significant reduction in VEGFR1 expression (Fig. 6C) and VE-cadherin expression (Fig. 6D). Thus, although VEGF-A is not sufficient to initiate HBMSC differentiation to ECs, it enhances EC differentiation in sustained high cell density cultures.

To confirm that VEGF-A stimulated VEGFR1 expression over 14 days, HBMSCs plated at high density were exposed to recombinant VEGF-A_._ Following culture of HBMSCs at high density in the presence of VEGF-A, VEGFR1 transcript (Fig. 6E) and protein expression (Fig. 6F) were significantly enhanced over this time frame. Thus, Notch-induced VEGF-A supports EC differentiation in sustained high cell density cultures.

### Tubule formation by HBMSCs precultured at high density

**Fig. 7:**
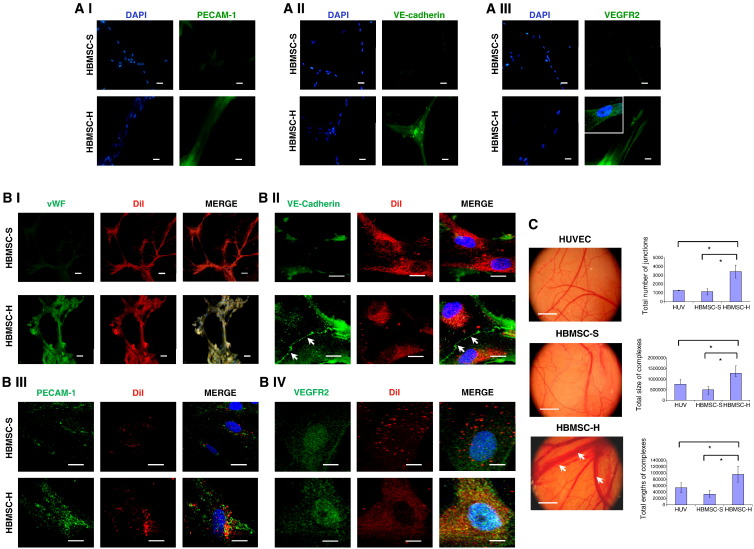
Expression of endothelial markers by high density HBMSCs on Matrigel and chick chorioallantoic (CAM) membranes. (A) To study the capacity of HBMSCs to form tubules, 20 000 HBMSCs cultured at high density (HBMSC-H) for 28 days, or cultured under standard conditions (HBMSC-S) for 7 days, were seeded onto growth factor reduced Matrigel in 0.5% serum DMEM for 48 h. (A I–III) Immunofluorescence analysis of (A I) PECAM-1, (A II) VE-cadherin, and (A III) VEGFR2; inset depicts an enlarged region of the image. All immunofluorescent images were taken using an Olympus BX51 widefield microscope (20x objective). Scale bars represent 20 μm. A representative of two independent experiments is shown in each case. (B) The effect of exposing HBMSCs to the angiogenic environment of the chick chorioallantoic membrane (CAM) was investigated. To identify implanted cells from those of the chick, 20 000 HBMSCs were prelabeled with DiI, seeded onto Matrigel-treated coverslips, and then placed in direct contact with the CAM of a Day 6 chick embryo for 48 h. (B I) Immunofluorescence analysis of vWF, using an Olympus BX51 widefield microscope (20x objective). vWF, green; DiI, red. Scale bars represent 100 μm. (B II–IV) Immunofluorescence analysis of (II) VE-cadherin, (III) PECAM-1, and (IV) VEGFR2, using a Nikon C1 upright confocal microscope (60x objective). White arrows indicate that VE-cadherin is present at the cell surface. Scale bars represent 20 μm. VE-cadherin, PECAM-1, or VEGFR2, green; DiI, red. (C) CAM vascularisation following exposure to HUVECs or HBMSC-coated coverslips for 48 h. White arrows indicate where the presence of HBMSCs precultured at high density (HBMSC-H) induced larger blood vessels in the CAM. The degree of vascularisation was quantified with Angioquant software measuring total number of junctions, total size of complexes, and total lengths of complexes. * *P* < 0.05. Ten representative images were taken for each treatment, using a Nikon stereomicroscope with each analysis performed in triplicate. Scale bars represent 6 mm. A representative of two independent experiments is shown in each case.

HBMSCs that had been precultured for up to 28 days at high cell density were cultured on Matrigel and exposed to chick chorioallantoic membranes (CAM). Both control and high density cultured HBMSCs readily formed widespread tubule networks on Matrigel, but only high density cultured HBMSCs expressed EC markers (Fig. 7A).

While exposure to the CAM induced control HBMSCs to express EC markers (vWF, VE-cadherin, PECAM-1, and VEGFR2), in comparison, HBMSCs cultured at high density produced more abundant EC marker expression, formed more robust tubules (Fig. 7B), and were found to induce significantly larger blood vessels in the CAM (Fig. 7C).

We also evaluated the ability for HBMSCs precultured at high density to retain their EC phenotype when subcultured at low cell density. HBMSCs cultured at high density for 28 days were trypsinised and subcultured at low cell density for 7 days, and then their level of VEGFR1 and vWF expression was determined by immunoblot analysis (Supplementary Fig. S4D). Compared to HBMSCs cultured at high density for 28 days, those subsequently subcultured at low density for 7 days expressed only slightly lower levels of VEGFR1 and vWF, suggesting that the EC phenotype was maintained.

Together, these data show that coordinated Notch and VEGF-A signals direct the differentiation of HBMSCs toward ECs.

## Discussion

**Figure 8:**
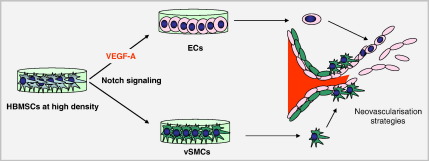
Model showing how Notch signaling and VEGF-A direct the vascular differentiation of cultured HBMSCs. HBMSCs plated and cultured at high density are induced to differentiate to an EC fate through Notch signaling and Notch-induced VEGF-A*.* These mechanisms may be therapeutically applicable for neovascularisation.

Defining the mechanisms that regulate HBMSC differentiation along vascular cell lineages will advance opportunities to exploit them in cell-based and neovascularisation therapies. While HBMSC differentiation toward vSMCs involves mechanical strain (Kurpinski et al., 2006), DNA methylation changes (Wakitani et al., 1995), and TGFβ and Notch signaling (Kurpinski et al., 2010), mechanisms underlying the differentiation of HBMSCs to ECs remain incompletely understood but largely attributed to VEGF-A. In this study, the inductive effect of cell contact has been identified as a potent trigger to vascular differentiation (Fig. 8). Cell contact-activated Notch signaling initially induced vascular progenitor cells with EC and vSMC characteristics. Subsequent differentiation toward ECs, but not vSMCs, was regulated by Notch-induced VEGF-A. Thus, coordinated Notch and VEGF-A signaling directs HBMSCs toward ECs.

Notch receptors are critical for vascular development and function (Gridley, 2007, Lawson et al., 2001, Boulton et al., 2008, Roukens et al., 2010, Jakobsson et al., 2010). In this study, Notch receptors 1–3 were directly implicated in the induction of vascular progenitor status in the HBMSCs cultured at high density, and thus contribute to vascular commitment. Although Notch 4 can be expressed in vascular cells (Wu et al., 2005b), we did not detect Notch 4 either at 24 h after plating at high density or by 28 days (not shown). Thus, Notch 4 appears not to be necessary for the differentiation of HBMSCs to ECs.

Density-dependent Notch signaling rapidly induced a vascular progenitor cell state characterised by expression of EC and vSMC markers. Since VEGFR1 is not endothelial-specific, despite being a major EC product (Supplementary Fig. S5), several other endothelial markers were utilized including VEGF-A, VE-cadherin, PECAM-1, and vWF. We found that VEGF-A was strongly induced by Notch, which in turn stimulated commitment of progenitor HBMSCs along the EC lineage. Interestingly, exposure to exogenous VEGF-A at standard density over 24 h was not sufficient to induce HBMSCs to express EC markers (Supplementary Fig. S4). Therefore, VEGF-A is not sufficient to initiate HBMSC differentiation to ECs, although it supports differentiation in sustained high density cultures. *In vivo* studies have documented how VEGF can disrupt vSMC function, ablating pericyte coverage and causing vessel destabilisation (Greenberg et al., 2008). Thus, the Notch-induced VEGF-A may have inhibited HBMSC differentiation to vSMCs while promoting HBMSC differentiation to ECs, and tubule formation.

We investigated several functional EC features of the high density HBMSCs. Uptake of LDL is a characteristic function of ECs, and here we showed that HBMSCs cultured at high density effectively took up LDL, implying an EC-like state. However, we cannot exclude that the increased LDL uptake might, in part, reflect fat accumulation. When the cells were plated on Matrigel or on CAM membranes, it was clear that they not only produced much larger tubules but also enhanced the expression of several EC markers. Taken together, these data indicate that the high density cells were committed to the EC lineage.

Our experiments indicate that low density culture suppresses the potential of HBMSCs to differentiate along vascular lineages. Therefore, a double-labeling immunofluorescence approach was used to address whether our HBMSC populations might be bipotential with the ability to differentiate along either SMC or EC lineages. Many cells were found to express both vSMC and EC markers, so it was clear that both lineages can be induced in the same cells over an initial 24 h after plating at high density. However, there may be some heterogeneous potential within our HBMSC populations.

HBMSCs are known to have therapeutic potential for tissue remodeling and repair. Being multipotent, they can not only differentiate along a range of lineages, they can also enhance tissue repair by secreting anti-inflammatory factors (Lee et al., 2009b). Their culture conditions can affect their phenotypic status; for example, culture in 3D spheres was recently shown to enhance the expression of anti-inflammatory molecules (Bartosh et al., 2010). In this study, we have demonstrated that it is possible to control their vascular differentiation *in vitro*, through regulating plating density and cell–cell contact. High density triggers EC differentiation by rapid induction of Notch signaling which in turn up-regulates VEGF-A, a growth factor that consolidates EC differentiation. These high density *in vitro* conditions may mirror extensive cell contact areas in EC monolayers *in vivo*. Our study, which has defined mechanisms of vascular differentiation of HBMSCs, provides new opportunities to regulate HBMSCs in therapeutic neovascularisation.

## Conclusion

This study has highlighted the coordinated role of cell density-dependent Notch signaling and Notch-induced VEGF-A in directing the vascular fate of HBMSCs.

## Materials and methods

### Antibodies

Primary antibodies used for immunoblot and immunofluorescence analysis were goat anti-human VEGFR1 (AF321), rabbit anti-human phospho-VEGFR1 (Y1213) (AF4170), and goat anti-human hairy enhancer of split-1 (HES-1) (AF3317), all from R&D Systems. Mouse anti-human von Willebrand factor (vWF) (3H3126) (sc-73267), mouse anti-human vascular cell adhesion molecule-1 (VCAM-1) (P3C4) (sc-20070), mouse anti-human phosphotyrosine (PY99) (sc-7020), rabbit anti-human smoothelin B (sc-28562), rabbit anti-human Notch 3 (m-134) (sc-5593), and goat anti-human Notch 4 (E-12) (sc-32613) were all from Santa-Cruz Biotechnology. Rabbit anti-human Notch 1 (C37C7), rabbit anti-human Notch 2 (8A1), mouse anti-human platelet/endothelial cell adhesion molecule-1 (PECAM-1) (89C2) (3528), rabbit anti-human vascular endothelial cadherin (VE-cadherin) (2158) and rabbit anti-human VEGFR2 (55B11) (2479) were all from Cell Signaling Technology. Mouse anti-human smooth muscle alpha actin (αSMA) (clone 1A4), mouse anti-human calponin (clone Calp), mouse anti-human smooth muscle myosin heavy chain 1 (SM-MHC-1) (clone SMMS-1), and negative control mouse IgG_1_ (X093101) were purchased from DAKO.

### Growth factors and inhibitors

VEGF-A_165_ (298-VS) and VEGF neutralising monoclonal antibody (clone 26503) were obtained from R&D Systems. VEGFR tyrosine kinase inhibitor (KRN633) was purchased from Calbiochem. The gamma secretase inhibitor *N*-[*N*-(3,5-difluorophenacetyl-L-alanyl)]-*S*-phenylglycine *t*-butyl ester (DAPT) was obtained from Sigma-Aldrich.

### Cell culture

Human bone marrow stromal cells were obtained from the bone marrow of a 28-year-old female and from a 21-year-old male (Lonza). The HBMSCs had a typical spindle-shaped morphology (Supplementary Fig. S1A), were positive for CD73, CD29, CD44, CD51, and CD105, but negative for the haematopoietic cell marker CD34 (Supplementary Fig. S1B), and had the potential to differentiate toward adipocyte, osteocyte, or chondrocyte lineages (Supplementary Fig. S1C). HBMSCs cultured on 0.1% gelatin (Sigma-Aldrich) were maintained in basal medium (Invitrogen) supplemented with 2% L-glutamine and 0.1% penicillin/streptomycin. HBMSCs were cultured at 37 °C in a humidified atmosphere of 20% O_2_, 5% CO_2_, and used at passage 4–5. Human umbilical vein endothelial cells from 43-year-old and 29-year-old females (Invitrogen) were maintained in endothelial cell basal medium (Lonza), containing singleQuot supplements, and used at passage 4. Human dermal fibroblasts (HDFs) from a 51-year-old male, obtained from the European Collection of Cell Cultures, were maintained in Dulbecco's minimal essential medium (DMEM) (Invitrogen) containing 10% fetal bovine serum, 1% L-glutamine, 0.2% penicillin/ streptomycin, and used at passage 8.

### Cell plating

For standard culture conditions, cells were seeded at 70 000 cells in 9.6 cm^2^ at initial plating (corresponding to 70% confluence). For high cell density experiments, HBMSCs were seeded at 100 000 cells in 9.6 cm^2^ at initial plating. For low density experiments, HBMSCs were seeded at 10 000 cells in 9.6 cm^2^ at initial plating.

### Semiquantitative and quantitative reverse-transcription polymerase chain reaction

Total RNA was isolated from cultured cells using Trizol reagent. Total RNA (1 μg) was reverse-transcribed and subjected to polymerase chain reaction (PCR). Quantitative PCR was performed using a SYBR green quantitative PCR core kit (Eurogentec), with each sample run in triplicate. Data were normalized to GAPDH, and relative expression was calculated according to the 2-[delta]CT formula. Oligonucleotide primers for PCR were designed using Primer3 software. Each primer pair was designed using the same parameters (70- to 100-bp product size and *T*_m_ of 60 °C). PCR were repeated using a second primer set (sequences not shown). Reaction products were resolved using 2.5% ultrapure agarose (Invitrogen) gel electrophoresis run at 100 V for 2 h in 1X TAE (Tris base, acetic acid, EDTA) buffer containing 2.5 μl Gel Red (Biotium), and visualised using a transilluminator.

### Primer sequences

Primer sequences were as follows: GAPDH (71 bp), forward (5′-AAGGGCATCCTGGGCTAC-3′) and reverse (5′-GTGGAGGAGTGGGTGTCG-3′); VEGFR1 (99 bp), forward (5′-GCGACGTGTGGTCTTACG-3′) and reverse (5′-GGCGACTGCAAAAGTCCT-3′); VEGFR-2 (81 bp), forward (5′-CATCCAGTGGGCTGATGA-3′) and reverse (5′-TGCCACTTCCAAAAGCAA–3′); VEGFR-3 (87 bp), forward (5′-GATGCGGGACCGTATCTG-3′) and reverse (5′-ATCCTCGGAGCCTTCCAC-3′); HES1 (91 bp), forward (5′-CCAAAGACAGCATCTGAGCA-3′) and reverse (5′-TCAGCTGGCTCAGACTTTCA-3′); VEGF-A (98 bp), forward (5′-CACCCATGGCAGAAGGAG-3′) and reverse (5′-CACCAGGGTCTCGATTGG-3′); VE-cadherin (74 bp), forward (5′-GGAGCCGAGCATGTGTCT-3′) and reverse (5′-TCTGCAAGGTGTGCCTGA-3′).

### Immunoblot analysis

Cells were washed with phosphate-buffered saline (PBS), incubated with lysis buffer for 15 min on ice, and then isolated. Total protein lysates were quantitated using a bicinchoninic acid (BCA) assay (Pierce), electrophoresed under reducing conditions using NuPAGE 4–12% Bis-Tris gels at 200 V for 2 h, and then transferred to a nitrocellulose membrane using a NuPAGE Western transfer system (Invitrogen). Membranes were blocked in 4% milk solids and then incubated with the primary antibody overnight at 4 °C (1:500–1:1000 dilution). Membranes were incubated with horseradish peroxidise (HRP)-conjugated secondary antibodies (DAKO, UK) for 2 h and then developed with SuperSignal West Dura Extended Duration Chemiluminescence (Thermo Fisher Scientific). Proteins were visualised using KODAK X-AR film and density of bands was determined using Gene Tools software (Syngene), and then the corresponding loading control was normalised.

### Immunofluorescence microscopy

After culturing cells on gelatin-coated 1.7 cm^2^ coverslips, cells were washed with PBS and fixed in 4% paraformaldehyde for 20 min, quenched in 0.2 M glycine (Fisher Scientific) for 20 min, and then permeabilised in 0.5% Triton X-100 (Sigma-Aldrich) for 4 min. Nonspecific binding was blocked with 2% fish skin gelatin solution (Sigma-Aldrich) for 1 h, and then cells were incubated overnight at 4 °C with primary antibodies (1:50–1:100 dilution) in blocking solution. After washing with PBS, cells were incubated with Alexafluor secondary antibodies (Alexa-Fluor 488 conjugated or Alexa-Fluor 546/555 conjugated (Invitrogen) (1:200 dilution) for 2 h at room temperature. For F-actin staining, cells were also incubated with rhodamine-conjugated phalloidin (Invitrogen) (1:500 dilution) for 2 h at room temperature. Cells were then washed and coverslips mounted using Prolong Gold Antifade solution containing 4′,6-diamidino-2-phenylindole (DAPI) (Invitrogen). At least 20 representative images of each sample were captured for each analysis. All immunofluorescence experiments included no primary antibody, no secondary antibody, and isotype-specific IgG_1_ controls to confirm specificity (data not shown).

For fluorescence microscopy, images were collected on an Olympus Widefield BX51 upright microscope using a 20x/UPlanFLN objective and captured using a Coolsnap HQ camera (Photometrics) through MetaVue Software (Molecular Devices). Specific band pass filter sets for DAPI, FITC, and tetramethylrhodaminoisothiocyanate (TRITC) were used to prevent bleed-through from one channel to the next. For confocal microscopy, images were collected using a Nikon C1 confocal on an upright 90i microscope with a 60x/1.40 Plan Apo objective and 3x confocal zoom. Images for DAPI, FITC, and Texas red were excited with the 405, 488, and 543 nm laser lines, respectively. Images were then processed and analysed using Nikon EZ-C1 FreeViewer v3.3 software. For Delta Vision microscopy, a Delta Vision restoration microscope (Applied Precision) 40x/0.85 UPlan Apo objective and Coolsnap (Photometrics) camera was utilised. Raw images were deconvolved using the Softworx software; maximum intensity projections of these deconvolved images are shown.

### Flow cytometry

Following trypsinisation, cells were left to recover in growth medium for 1 h, washed twice in wash buffer (0.5% bovine serum albumin in PBS) and 1 × 10^5^ cells incubated with either phycoerythrin (PE) conjugated or unconjugated primary antibody for 1 h, and then washed three times with wash buffer. For an unconjugated antibody, cells were additionally incubated with a species-specific fluorescein isothiocyanate (FITC)-conjugated secondary antibody (1:200) (Dako) for 45 min and then washed three times with wash buffer. For each analysis, 100 000 cells were counted using a FACscan cytometer at a flow rate less than 200 events per second.

### VEGF-A detection

Enzyme-linked immunosorbent (ELISA) assays were performed using the Quantikine human VEGF-A immunoassay (R&D Systems), according to the manufacturer's protocol.

### Notch activation

Notch was activated either by ethylenediaminetetraacetic acid (EDTA) (Rand et al., 2000) or recombinant Jagged-1. For EDTA experiments, cells were stimulated for 15 min with 5 mM EDTA, washed twice with basal medium, and left to recover for 24 h, before isolation. For Notch activation using Jagged-1, recombinant human Jagged-1/Fc chimera (1277-JG) (R&D Systems) was immobilised on culture plates by incubating plates with a solution of Jagged-1 (5 μg/ml) in PBS for 2 h at 37 °C. Control plates did not contain recombinant human Jagged-1. For these experiments, HBMSCs were seeded onto plates at low or high density and cultured for 24 h.

### HBMSC differentiation assay

HBMSCs were induced to differentiate toward adipocyte, osteocyte, or chondrocyte lineages using an MSC functional identification kit (R&D Systems), according to the manufacturer's protocol, and then cells were prepared for immunofluorescence analysis as previously described using the antibodies provided. In addition, lipid droplets were stained using boron-dipyrromethene (Bodipy) 493/503 (Invitrogen) and F-actin filaments stained using phalloidin.

### siRNA knockdown transfections

HBMSCs (5 × 10^5^ cells), together with 3 μg small interfering RNAs (siRNAs), were transfected by electroporation using a human Nucleofector kit (Lonza), and cultured overnight in growth medium. Validated siRNAs, which were functionally tested to provide ≥ 70% target gene knockdown, were used for VEGF-A knockdown (Qiagen). For Notch knockdowns, siRNAs for Notch1 (sc-36095), Notch2 (sc-40135), and Notch3 (sc-37135) (Santa Cruz, USA) were employed, with scrambled siRNA as a control (Qiagen).

### Immunoprecipitation analysis

Cell lysates were precleared using 10% (w/v) protein A-Sepharose (GE Healthcare), and then precleared lysates were incubated with primary antibody overnight at 4 °C. Immune complexes were isolated by incubation with 10% (w/v) protein A-Sepharose for 2 h at 4 °C. Protein A-Sepharose beads were washed three times using ice-cold lysis buffer, and then resuspended in NuPAGE gel loading buffer (Invitrogen).

### Endothelial functional tests

#### LDL uptake assay

ECs readily take up 1,1′-dioctadecyl-3,3,3′,3′-tetramethylindocarbocyanine-labeled acetylated low density lipoprotein (Dil-Ac-LDL), where the lipoprotein is degraded by lysosomes and fluorescent Dil accumulates within intracellular membranes. Cells were incubated with 10 μg/ml Dil-Ac-LDL (BT-906) (Biomedical Technologies Ltd) for 3 h at 37 °C, washed with PBS, fixed in 4% paraformaldehyde for 20 min, and then quenched in 0.2 M glycine for 20 min. Uptake of DiI-ac-LDL was assessed by mounting coverslips using Prolong Gold Antifade solution containing DAPI (Invitrogen) and examination using an Olympus Widefield BX51 microscope.

#### Induction of vascular cell adhesion molecule-1 in response to TNFα

Recombinant human tumour necrosis factor α (TNFα) (10 ng/ml) (R&D Systems; 210-TA-010) was added to cells for 24 h, and then cells were prepared for immunofluorescence analysis as previously described.

#### Matrigel tubule formation assay

A thin layer of growth factor-reduced Matrigel (BD Biosciences) was used to coat round glass coverslips and allowed to set for 30 min at 37 °C. Cells (20 000) in 0.5% serum containing DMEM were seeded onto the surface of Matrigel and incubated at 37 °C for up to 48 h to allow tubule formation, and then prepared for immunofluorescence analysis as previously described.

#### Chorioallantoic membrane (CAM) angiogenesis assay

Fertilized white chicken eggs were incubated for 6 days at 37.5 °C and 43% humidity in a specialised egg incubator. To distinguish implanted cells from chick cells on the CAM, implanted cells were prelabeled with 10 μg/ml 1,1-dioctadecyl-3,3,3,3-tetramethylindocarbocyanine perchlorate DiI (Sigma-Aldrich) for 30 min at 37 °C, seeded onto Matrigel-coated coverslips, and then left to adhere for 1 h. Eggs were cleaned with 70% ethanol and a square window was cut into the shell with dissecting scissors to reveal the underlying embryo and CAM blood vessels. The membrane covering the CAM surface was carefully removed with forceps and coverslips were implanted cell face down onto the CAM. The window was sealed with transparent tape and the egg was returned to the incubator for 48 h at 37.5 °C and 65% humidity, after which coverslips were removed and prepared for immunofluorescence as previously described.

### Statistical analysis

In all quantitation experiments, results were expressed as the mean ± standard deviation. Statistical differences between sets of data were determined using a paired *t* test on SigmaPlot 8.0 software, with *P* < 0.05 considered significant.

## Appendix A Supplementary data

Fig. S1. HBMSC characterisation. (A) Representative phase contrast image of HBMSCs plated at 70% confluence (standard culture), showing characteristic spindle-shaped morphology. Image was obtained using an Olympus (CK X41) microscope (20× objective). Scale bar = 200 μm. (B) Flow cytometry of HBMSCs plated at 70% confluency and cultured for 48 hours. HBMSCs were shown to express characteristic cell surface markers CD73 (ecto 5′ nucleotidase), CD29 (integrin β1 chain), CD44 (hyaluronan receptor), CD105 (endoglin) and CD51 (αV integrin), but did not express the haematopoietic marker (CD34). Red peaks denote PE-conjugated antibodies; green peaks denote FITC conjugated antibodies, while black peaks represent a control IgG_1_ antibody. Data are representative of two independent experiments for each analysis. (C) HBMSCs could be induced to differentiate towards adipogenic, osteogenic or chondrogenic lineages. HBMSCs were induced to differentiate towards (I) adipogenic, (II) osteogenic or (III) chondrogenic lineages by using defined differentiation media over 14 days, then analysed by immunofluorescence microscopy. Un-treated HBMSC controls were cultured in basal media over 14 days. Immunofluorescence analysis of (I) adipogenic differentiation showing Bodipy 493/503 (green) and FABP-4 (red), (II) osteogenic differentiation showing osteocalcin (green) and F-actin (red), and (III) chondrogenic differentiation showing aggrecan (green) and F-actin (red). Images were obtained using a Nikon C1 upright confocal microscope (60× objective). Nuclei were stained with DAPI (blue). Scale bars = 20 μm. Representative images are from two independent experiments. Fig. S2. HBMSCs expressed Notch receptors 1, 2 and 3. (A-F) Immunofluorescence analysis of Notch receptors 1–3 in HBMSCs cultured at low (HBMSC-L) or high density (HBMSC-H) for 24 hours. Notch receptors (green), DAPI (blue). Images were taken using a Nikon C1 upright microscope (60× objective). Representative images obtained from two independent experiments. (G, H) show IgG_1_ negative controls. Scale bars = 20 μm. Fig. S3. Notch signaling controls. (A) As a control for DAPT treatment, HES1 transcript levels were determined by RT-PCR, following 50 μM DAPT treatment in HBMSCs cultured at low (L) or high density (H) for 24 hours. GAPDH was used as loading controls. (B) siRNA knockdown of Notch receptors. Immunoblot analysis of Notch receptor 1 (N1), Notch receptor 2 (N2) and Notch receptor 3 (N3) protein expression in HBMSCs cultured for 24 hours at high density, following transfection with 3 μg scrambled (Scr) siRNA as controls (lanes 1, 3 and 5 respectively) or validated target siRNAs for Notch receptors 1, 2 or 3 (lanes 2, 4 and 6 respectively). Membranes were re-probed with an alternative Notch receptor as loading controls and to validate the specificity of each Notch receptor siRNA. (C) As a control for EDTA treatment, HES1 transcript levels were determined by RT-PCR, following 5 mM EDTA treatment in HBMSCs cultured at high density (H) for 24 hours. GAPDH was used as loading controls. VEGFR signaling controls. (D) To verify the efficacy of the VEGF-A neutralisation antibody, HUVECs were either un-treated (Cont), or treated with 1 μg/ml anti-VEGF neutralising antibody (VEGF-I), for 15 min in 0.5% serum DMEM, then stimulated with 50 ng/ml VEGF-A. VEGFR2 was isolated by immunoprecipitation using an anti-VEGFR2 antibody, then tyrosine phosphorylation detected by immunoblot analysis using an anti phosphotyrosine antibody. Membranes were re-probed with an antibody to total VEGFR2 for loading controls. (E-F) To establish the efficiency of VEGF-A knockdown, the level of secreted VEGF-A and the abundance of VEGF-A transcript were determined following knockdown. (E) ELISA assay of VEGF-A derived from medium taken from scrambled siRNA control (Scr ↓) or knockdown VEGF-A transfected HBMSCs, normalised against corresponding β-actin levels. * represents p < 0.05 compared to scrambled control HBMSCs. (F) RNA was isolated from scrambled control (S) or knockdown VEGF-A (V) HBMSCs, then semi-quantitative RT-PCR analysis used to detect GAPDH (lanes 1 and 2) and VEGF-A (lanes 3 and 4) transcript expression. (G) To determine the effectiveness of the chemical inhibitor of VEGF signaling, HUVECs were either un-treated (Cont), or treated with 0.5 μM VEGF receptor tyrosine kinase inhibitor (VEGFR-I) for 30 min. VEGFR1 was isolated by immunoprecipitation using an anti-VEGFR1 antibody, and tyrosine phosphorylation was detected by immunoblot analysis using an anti-phosphoVEGFR1 antibody. Membranes were re-probed with an antibody to total VEGFR1 as a loading control. Fig. S4. Exposure to recombinant VEGF-A for 24 hours did not induce HBMSCs to express EC markers. (A-C) To determine whether exposure to exogenous VEGF-A alone over 24 hours could induce HBMSCs to express the EC markers (A) PECAM-1, (B) VEGFR1 or (C) VE-cadherin, HBMSCs cultured in standard conditions were stimulated with 50 ng/ml VEGF-A_165_ for 24 hours (HBMSC+) (lane 3) or un-stimulated (HBMSC-) (lane 2). HUVECs in standard culture conditions were used as a positive control (lane 1). β-actin was used as loading controls. Representatives of two independent experiments are shown for each analysis. While HUVECs were shown to express PECAM-1 (A; lane 1), VEGFR1 (B; lane 1) and VE-cadherin (C; lane 1), these EC markers could not be detected in HBMSCs cultured in either standard conditions (lane 2), or medium supplemented with 50 ng/ml VEGF-A_165_ for 24 hours (lane 3). HBMSCs cultured at high density retained their expression of EC markers when sub-cultured at low density. (D) The ability of HBMSCs cultured at high density to maintain their EC phenotype when sub-cultured at low cell density was evaluated, by determining the expression levels of VEGFR1 and vWF. Immunoblot analysis of VEGFR1 and vWF protein levels in HBMSCs cultured at high density for 28 days (lane 1) or following trypsinisation and sub-culture at low cell density for 7 days (lane 2). A representative of two independent experiments is shown. Fig. S5. HBMSCs plated at high cell density induced expression of VEGFR1, vWF and VE-cadherin. (A) VEGFR expression was examined by semi-quantitative RT-PCR analysis. HUVECs plated at standard cell density, and HBMSCs at low density (HBMSC-L) or high cell density (HBMSC-H), were cultured for 14 days, then semi-quantitative RT-PCR analysis used to detect VEGFR1 (99-bp) (lane 1); VEGFR2 (81-bp); (lane 2) and VEGFR3 (87-bp) (lane3) transcript expression. Two different primer pairs for VEGFRs 1–3 gave similar results, and GAPDH (71-bp) was used as a loading control. While all three VEGFR transcripts were expressed by HUVECs, no VEGFR transcripts were detected in HBMSCs cultured at low density, but VEGFR1 was expressed by HBMSCs cultured at high density. (B) VE-cadherin expression was examined by semi-quantitative RT-PCR analysis. HUVECs (HUV) plated at standard cell density and HBMSCs at low density (HBMSC-L) were cultured for 7 days, while HBMSCs at high cell density (HBMSC-H) were cultured for 28 days, then semi-quantitative RT-PCR analysis used to detect VE-cadherin (74-bp) (lanes 1-4) transcript expression. GAPDH (71-bp) was used as a loading control. Both HUVECs (lane 1) and HBMSCs at high density (lanes 2 and 3) were shown to express VE-cadherin, but no VE-cadherin transcript was detected in HBMSCs cultured at low density (lane 4). (C-D) Quantitative PCR (qPCR) analysis of VEGFR1 and vWF transcript levels in HBMSCs cultured at low or high density. HBMSCs were plated at low density (HBMSC-L) or high density (HBMSC-H) and cultured for 14 days, then qPCR analysis used to determine the levels of VEGFR1 and vWF transcripts. Each sample was run in triplicate. * represents p<0.05 compared to HBMSCs culture at low density. Data were normalized to GAPDH, and relative expression was calculated according to the 2-[Delta]CT formula. Compared to low density HBMSC culture, the levels of both VEGFR1 and vWF transcripts were significantly increased by culture at high density.

## References

[bb0005] Al-Khaldi A., Eliopoulos N., Martineau D., Lejeune L., Lachapelle K., Galipeau J. (2003). Postnatal bone marrow stromal cells elicit a potent VEGF-dependent neoangiogenic response in vivo. Gene Ther..

[bb0010] Aster J.C., Simms W.B., Zavala-Ruiz Z., Patriub V., North C.L., Blacklow S.C. (1999). The folding and structural integrity of the first LIN-12 module of human Notch1 are calcium-dependent. Biochemistry.

[bb0015] Augello, A., De Bari, C., 2010. The regulation of differentiation in mesenchymal stem cells. Hum. Gene Ther. 10, 1226–1238. 10.1089/hum.2010.173 20804388

[bb0020] Bai K., Huang Y., Jia X., Fan Y., Wang W. (2009). Endothelium oriented differentiation of bone marrow mesenchymal stem cells under chemical and mechanical stimulations. J. Biomech..

[bb0025] Ball S.G., Shuttleworth C.A., Kielty C.M. (2004). Direct cell contact influences bone marrow mesenchymal stem cell fate. Int. J. Biochem. Cell Biol..

[bb0030] Ball S.G., Shuttleworth C.A., Kielty C.M. (2007). Vascular endothelial growth factor can signal through platelet-derived growth factor receptors. J. Cell Biol..

[bb0035] Bartosh T.J., Ylöstalo J.H., Mohammadipoor A., Bazhanov N., Coble K., Claypool K., Lee R.H., Choi H., Prockop D.J. (2010). Aggregation of human mesenchymal stromal cells (MSCs) into 3D spheroids enhances their antiinflammatory properties. Proc. Natl Acad. Sci. USA.

[bb0040] Boulton M.E., Cai J., Grant M.B. (2008). Gamma-Secretase: a multifaceted regulator of angiogenesis. J. Cell. Mol. Med..

[bb0045] Casiraghi, F., Noris, M., Remuzzi, G., 2010. Immunomodulatory effects of mesenchymal stromal cells in solid organ transplantation. Curr. Opin. Organ Transplant. 10.1097/MOT.0b013e328340172c 20881495

[bb0050] Chen M.Y., Lie P.C., Li Z.L., Wei X. (2009). Endothelial differentiation of Wharton's jelly-derived mesenchymal stem cells in comparison with bone marrow-derived mesenchymal stem cells. Exp. Hematol..

[bb0055] Chung N., Jee B.K., Chae S.W., Jeon Y.W., Lee K.H., Rha H.K. (2009). HOX gene analysis of endothelial cell differentiation in human bone marrow-derived mesenchymal stem cells. Mol. Biol. Rep..

[bb0060] Greenberg J.I., Shields D.J., Barillas S.G., Acevedo L.M., Murphy E., Huang J., Scheppke L., Stockmann C., Johnson R.S., Angle N., Cheresh D.A. (2008). A role for VEGF as a negative regulator of pericyte function and vessel maturation. Nature.

[bb0065] Gridley T. (2007). Notch signaling in vascular development and physiology. Development.

[bb0070] Jakobsson L., Franco C.A., Bentley K., Collins R.T., Ponsioen B., Aspalter I.M., Rosewell I., Busse M., Thurston G., Medvinsky A., Schulte-Merker S., Gerhardt H. (2010). Endothelial cells dynamically compete for the tip cell position during angiogenic sprouting. Nat. Cell Biol..

[bb0075] Kurpinski K., Park J., Thakar R.G., Li S. (2006). Regulation of vascular smooth muscle cells and mesenchymal stem cells by mechanical strain. Mol. Cell. Biomech..

[bb0080] Kurpinski K., Lam H., Chu J., Wang A., Kim A., Tsay E., Agrawal S., Schaffer D., Li S. (2010). TGF-beta and Notch signaling mediate stem cell differentiation into smooth muscle cells. Stem Cells.

[bb0085] Lawson N.D., Scheer N., Pham V.N., Kim C.H., Chitnis A.B., Campos-Ortega J.A., Weinstein B.M. (2001). Notch signaling is required for arterial-venous differentiation during embryonic vascular development. Development.

[bb0090] Lee R.H., Pulin A.A., Seo M.J., Kota D.J., Ylostalo J., Larson B.L., Semprun-Prieto L., Delafontaine P., Prockop D.J. (2009). Intravenous hMSCs improve myocardial infarction in mice because cells embolized in lung are activated to secrete the anti-inflammatory protein TSG-6. Cell Stem Cell.

[bb0095] Lee R.H., Pulin A.A., Seo M.J., Kota D.J., Ylostalo J., Larson B.L., Semprun-Prieto L., Delafontaine P., Prockop D.J. (2009). Intravenous hMSCs improve myocardial infarction in mice because cells embolized in lung are activated to secrete the anti-inflammatory protein TSG-6. Cell Stem Cell.

[bb0100] Lozito T.P., Kuo C.K., Taboas J.M., Tuan R.S. (2009). Human mesenchymal stem cells express vascular cell phenotypes upon interaction with endothelial cell matrix. J. Cell. Biochem..

[bb0105] McBeath R., Pirone D.M., Nelson C.M., Bhadriraju K., Chen C.S. (2004). Cell shape, cytoskeletal tension, and RhoA regulate stem cell lineage commitment. Dev. Cell.

[bb0110] Ohata E., Tadokoro R., Sato Y., Saito D., Takahashi Y. (2009). Notch signal is sufficient to direct an endothelial conversion from non-endothelial somitic cells conveyed to the aortic region by CXCR4. Dev. Biol..

[bb0115] Oswald J., Boxberger S., Jorgensen B., Feldmann S., Ehninger G., Bornhauser M., Werner C. (2004). Mesenchymal stem cells can be differentiated into endothelial cells in vitro. Stem Cells.

[bb0120] Pittenger M.F., Mackay A.M., Beck S.C., Jaiswal R.K., Douglas R., Mosca J.D., Moorman M.A., Simonetti D.W., Crai S., Marshak D.R. (1999). Multilineage potential of adult human mesenchymal stem cells. Science.

[bb0125] Rand M.D., Lindblom A., Carlson J., Villoutreix B.O., Stenflo J. (1997). Calcium binding to tandem repeats of EGF-like modules. Expression and characterization of the EGF-like modules of human Notch-1 implicated in receptor-ligand interactions. Protein Sci..

[bb0130] Rand M.D., Grimm L.M., Artavanis-Tsakonas S., Patriub V., Blacklow S.C., Sklar J., Aster J.C. (2000). Calcium depletion dissociates and activates heterodimeric notch receptors. Mol. Cell. Biol..

[bb0135] Roukens M.G., Alloul-Ramdhani M., Baan B., Kobayashi K., Peterson-Maduro J., van Dam H., Schulte-Merker S., Baker D.A. (2010). Control of endothelial sprouting by a Tel-CtBP complex. Nat. Cell Biol..

[bb0140] Siekmann A.F., Covassin L., Lawson N.D. (2008). Modulation of VEGF signaling output by the Notch pathway. Bioessays.

[bb0145] Wakitani S., Saito T., Caplan A.I. (1995). Myogenic cells derived from rat bone marrow mesenchymal stem cells exposed to 5-azacytidine. Muscle Nerve.

[bb0150] Wu X., Huang L., Zhou Q., Song Y., Li A., Jin J., Cui B. (2005). Mesenchymal stem cells participating in ex vivo endothelium repair and its effect on vascular smooth muscle cells growth. Int. J. Cardiol..

[bb0155] Wu J., Iwata F., Grass J.A., Osborne C.S., Elnitski L., Fraser P., Ohneda O., Yamamoto M., Bresnick E.H. (2005). Molecular determinants of NOTCH4 transcription in vascular endothelium. Mol. Cell. Biol..

[bb0160] Xu J., Liu X., Chen J., Zacharek A., Cui X., Savant-Bhonsale S., Liu Z., Chopp M. (2009). Simvastatin enhances bone marrow stromal cell differentiation into endothelial cells via notch signaling pathway. Am. J. Physiol. Cell Physiol..

[bb0165] Yue W.M., Liu W., Bi Y.W., He X.P., Sun W.Y., Pang X.Y., Gu X.H., Wang X.P. (2008). Mesenchymal stem cells differentiate into an endothelial phenotype, reduce neointimal formation, and enhance endothelial function in a rat vein grafting model. Stem Cells Dev..

[bb0170] Zhang G., Zhou J., Fan Q., Zheng Z., Zhang F., Liu X., Hu S. (2008). Arterial-venous endothelial cell fate is related to vascular endothelial growth factor and Notch status during human bone mesenchymal stem cell differentiation. FEBS Lett..

