# Supplementary figures and images for: Density of human bone marrow stromal cells regulates commitment to vascular lineages
Source: Stem Cell Res. 2011 May;6(3):238–50. doi: 10.1016/j.scr.2011.02.001 (PMC3223522; doi:10.1016/j.scr.2011.02.001)

Figure S1

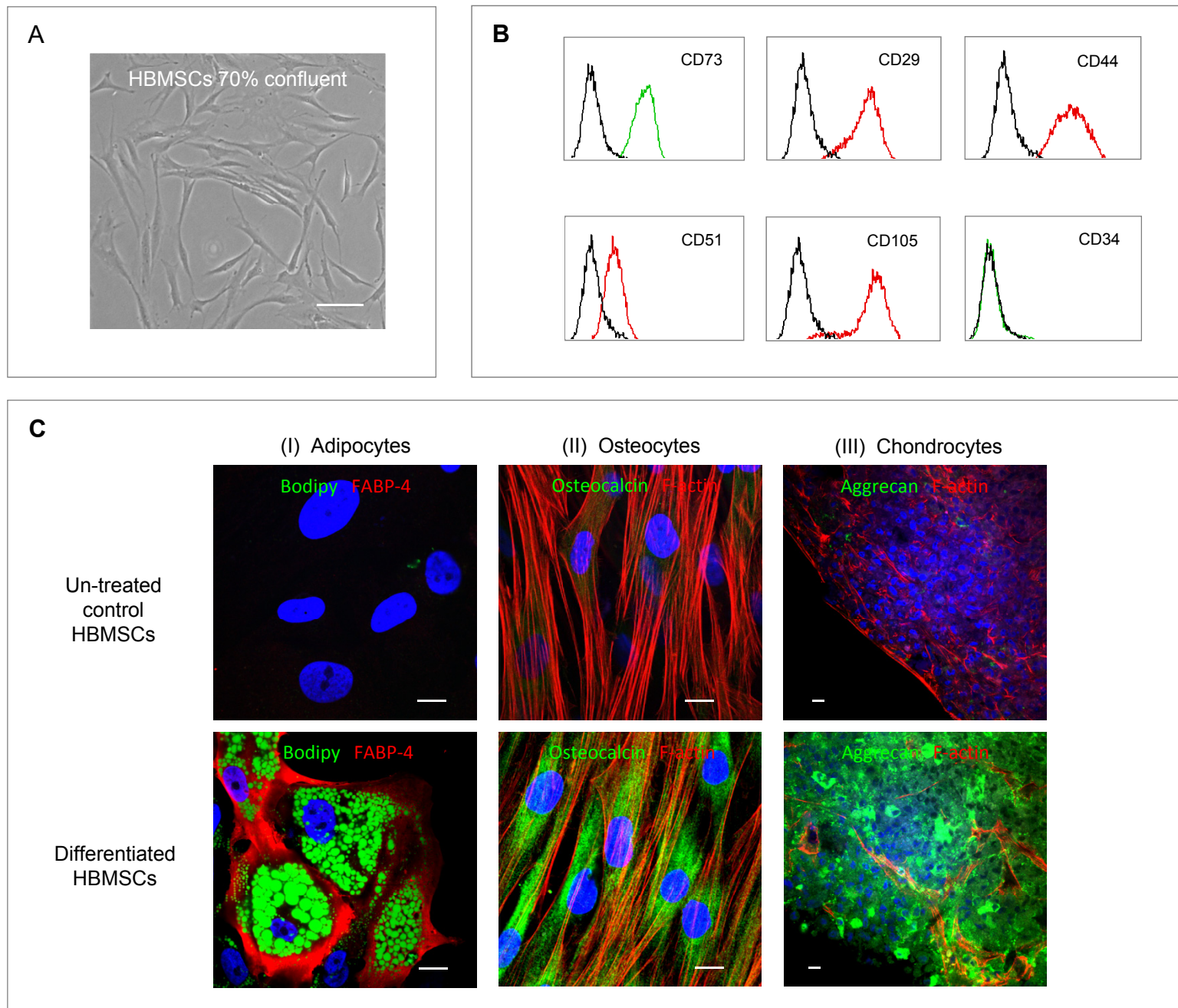

Figure S2

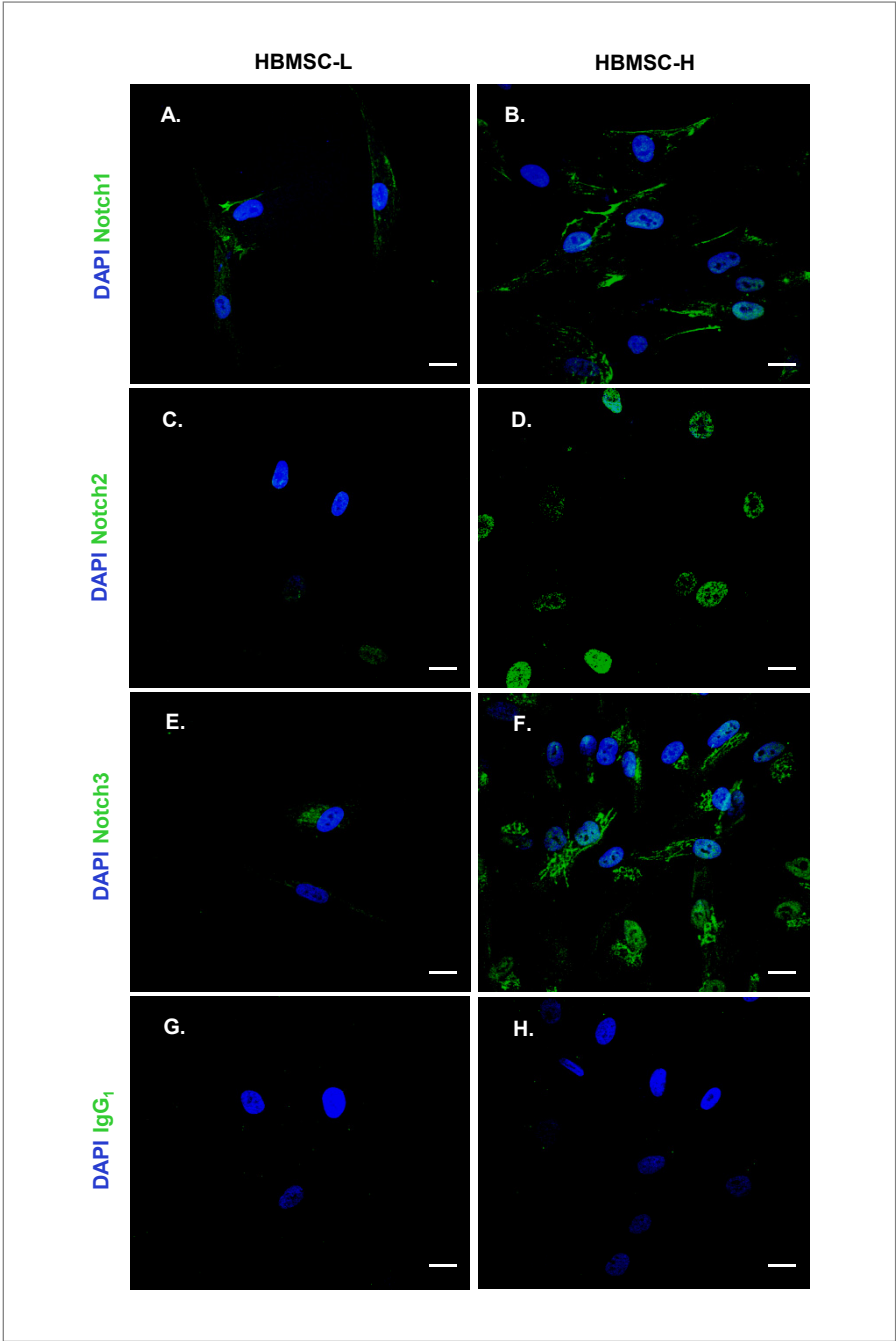

Figure S3

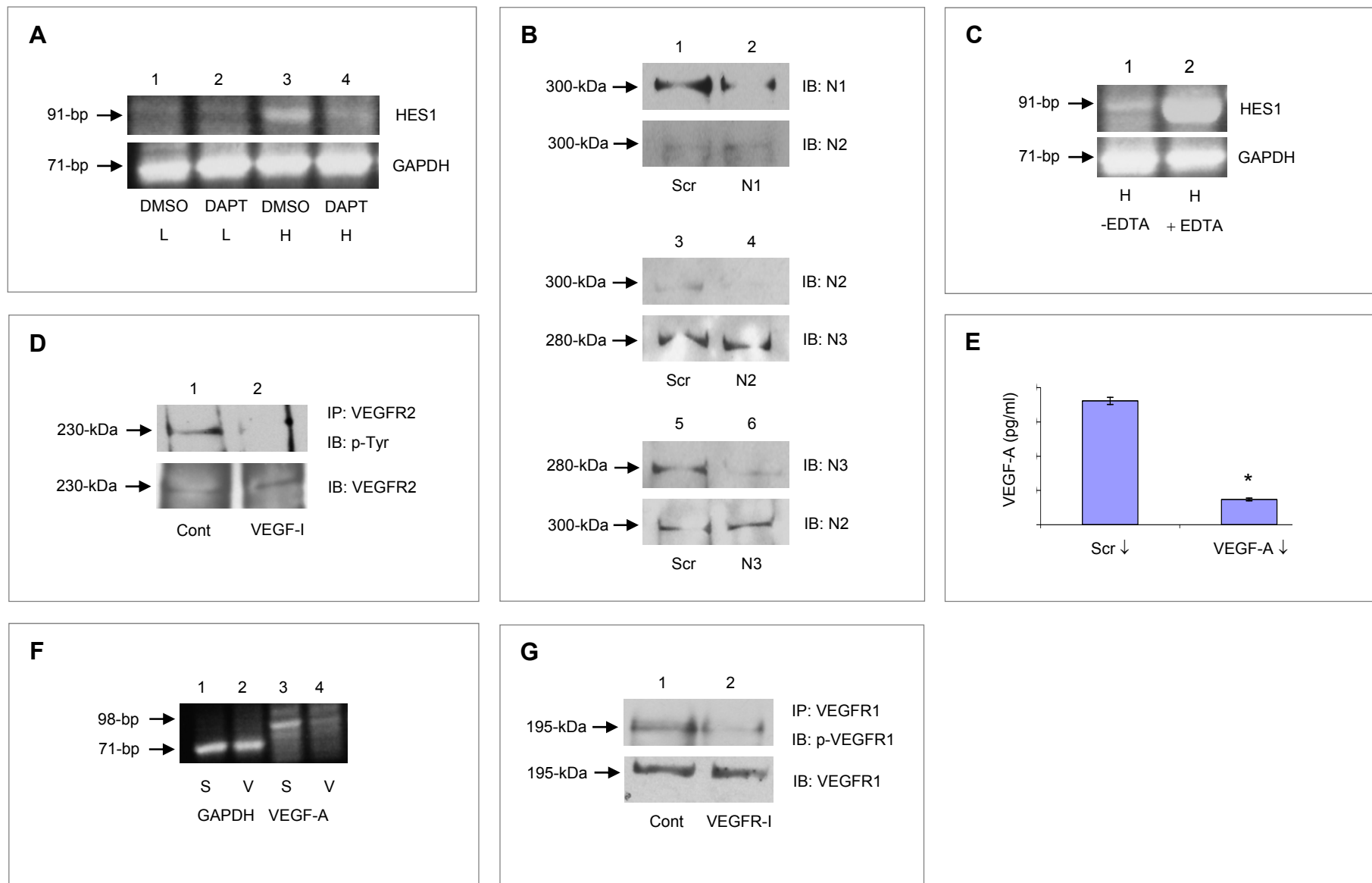

Figure S4

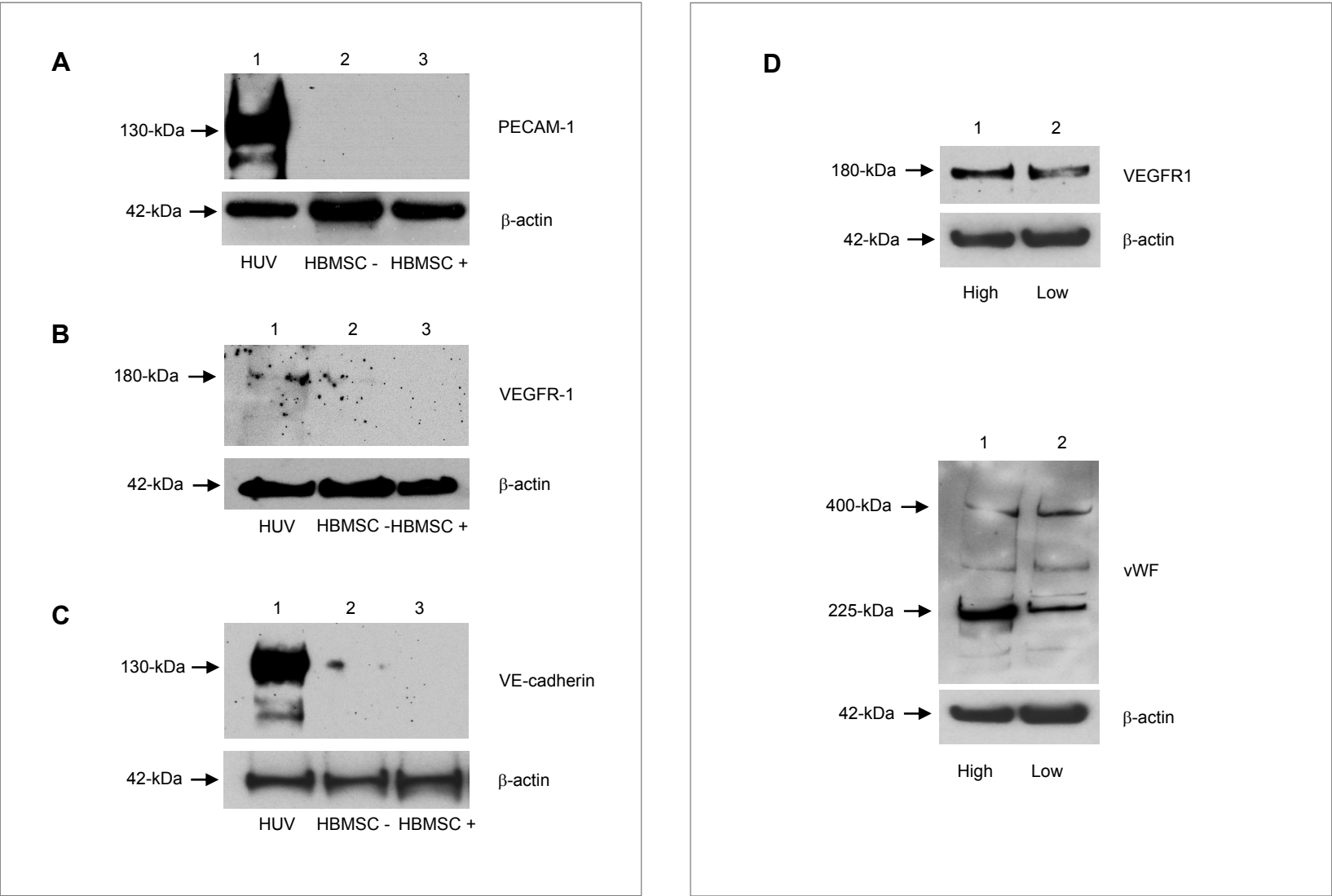

Figure S5

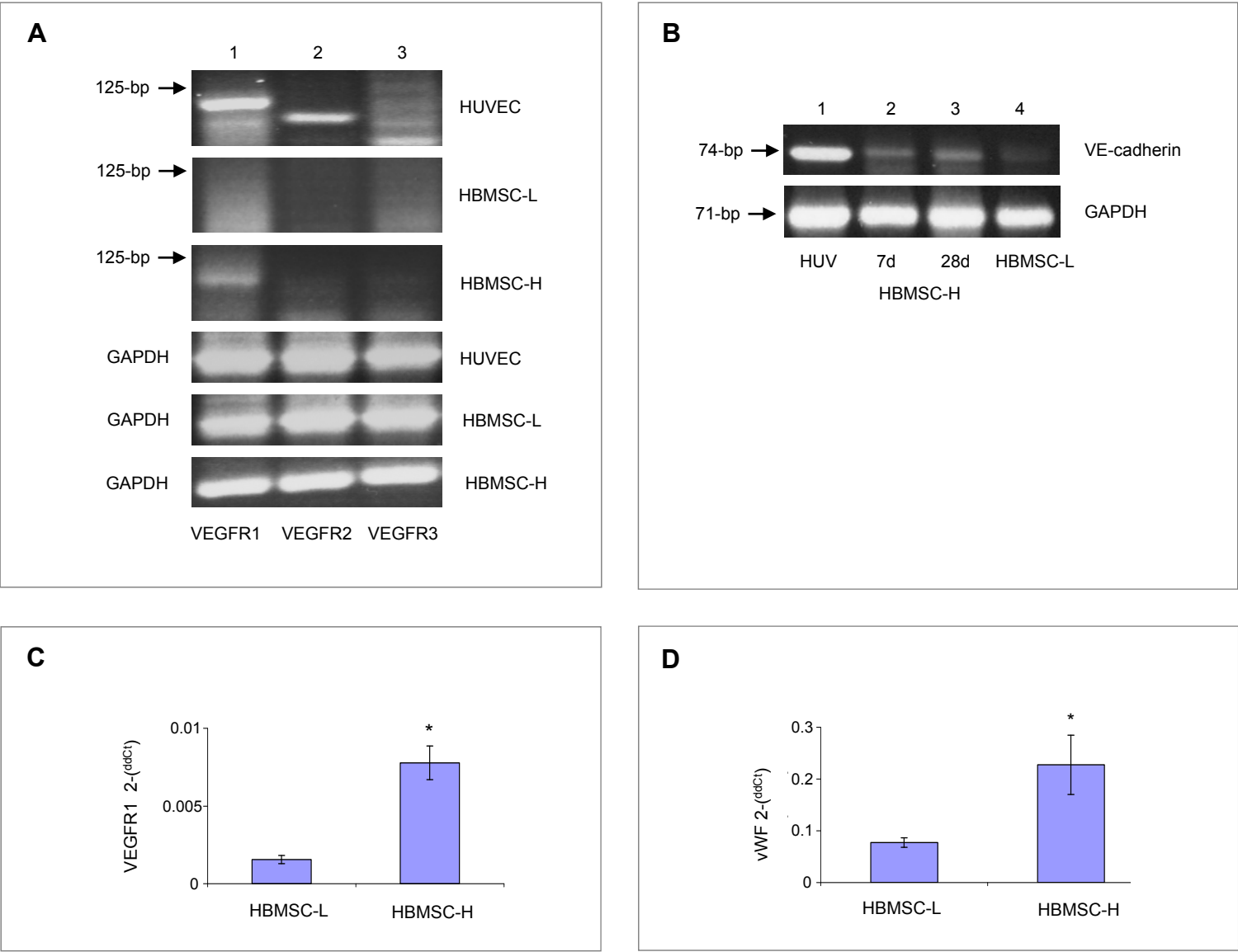

Supplement: Supplementary file 1 — Fig. S1. HBMSC characterisation. (A) Representative phase contrast image of HBMSCs plated at 70% confluence (standard culture), showing characteristic spindle-shaped morphology. Image was obtained using an Olympus (CK X41) microscope (20× objective). Scale bar = 200 μm. (B) Flow cytometry of HBMSCs plated at 70% confluency and cultured for 48 hours. HBMSCs were shown to express characteristic cell surface markers CD73 (ecto 5′ nucleotidase), CD29 (integrin β1 chain), CD44 (hyaluronan receptor), CD105 (endoglin) and CD51 (αV integrin), but did not express the haematopoietic marker (CD34). Red peaks denote PE-conjugated antibodies; green peaks denote FITC conjugated antibodies, while black peaks represent a control IgG1 antibody. Data are representative of two independent experiments for each analysis. (C) HBMSCs could be induced to differentiate towards adipogenic, osteogenic or chondrogenic lineages. HBMSCs were induced to differentiate towards (I) adipogenic, (II) osteogenic or (III) chondrogenic lineages by using defined differentiation media over 14 days, then analysed by immunofluorescence microscopy. Un-treated HBMSC controls were cultured in basal media over 14 days. Immunofluorescence analysis of (I) adipogenic differentiation showing Bodipy 493/503 (green) and FABP-4 (red), (II) osteogenic differentiation showing osteocalcin (green) and F-actin (red), and (III) chondrogenic differentiation showing aggrecan (green) and F-actin (red). Images were obtained using a Nikon C1 upright confocal microscope (60× objective). Nuclei were stained with DAPI (blue). Scale bars = 20 μm. Representative images are from two independent experiments. Fig. S2. HBMSCs expressed Notch receptors 1, 2 and 3. (A-F) Immunofluorescence analysis of Notch receptors 1–3 in HBMSCs cultured at low (HBMSC-L) or high density (HBMSC-H) for 24 hours. Notch receptors (green), DAPI (blue). Images were taken using a Nikon C1 upright microscope (60× objective). Representative images obtained from tw [file mmc1.pdf]
